# Changing the culture of chaos

**DOI:** 10.1111/aas.14563

**Published:** 2024-12-23

**Authors:** Martin I. Sigurdsson

**Affiliations:** ^1^ Faculty of Medicine University of Iceland Reykjavik Iceland; ^2^ Department of Anesthesiology and Critical Care Medicine Landspitali University Hospital Reykjavik Iceland

Many of the current practices in anaesthesia and intensive care are deeply rooted in culture, including institutional and regional culture built and maintained by influential clinicians. When current practice is compared between institutions and countries, the utilization of culture and local best practice in lieu of practices built on international guidelines based on high‐quality data can therefore sometimes appear to be random.

This is demonstrated in the multinational survey by Ellekjaer et al., comparing the use of different buffers in balanced crystalloid solutions.^1^ The results reveal that the utilization of solutions buffered with either lactate or acetate was roughly even; most clinicians surveyed were in favour of participation in a randomized trial comparing the two buffers, although the clinical importance of such study did not receive a high score. Such surveys are important as they highlight future research areas and can also be used to gauge the interest and perceived importance of research questions, maximizing the benefit and use of resources devoted to performing randomized studies. A limitation to the overall low response rate is the risk that the more polarized views are inflated.

Many well‐done large‐scale multicentre studies have been performed to answer important and clearly defined questions regarding nuances of intensive care practice. Amongst those are the several trials attempting to identify the ideal oxygen concentration provided to critically ill patients.^2^, ^3^, ^4^ While the trials have largely shown similar outcomes between the two arms, it is possible that there is either a difference that is small enough or hidden in a subgroup of the patients that results that is very challenging to demonstrate in a randomized trial given the complex and costly logistics of such trials. Such differences could however be important given the large and increasing number of patients receiving intensive care.

There are examples where other approaches have been utilized to answer some of these questions that are important but are logistically extremely challenging to answer in a randomized trial. Amongst those are pragmatic crossover trials, where local practice is changed in a protocolized manner, after careful consultation with local ethic committee and careful monitoring, given that informed consent is waived. Such trials have been used to show a small difference in major kidney events for critically ill patients based on choice of crystalloid resuscitation fluid^5^ and support equipotence of two oxygen saturation targets in ventilated patients.^6^

Similarly, ‘natural experiments’ occur when large scale‐changes are made to local practice during drug shortages that are common and predominantly affect medications that are rarely studied in industry‐sponsored trials. Example of this method is the demonstration of worse outcomes for sepsis patients during the period where noradrenaline shortage resulted in a rapid change towards phenylephrine and dopamine.^7^

Finally, the large rise in the conduct of platform trials in the recent years gives hope, because this platform allows studying multiple interventions in a single condition simultaneously. With the appropriate design, methodological support and multisite commitment to the trial, these trials allow for a remarkably efficient study design that allows simultaneous assessments of multiple interventions, maximizing the use of resources devoted to conducting high‐quality care. It is the hope that participation in these trials will become globally accepted, both on the academic and clinical levels.

## FUNDING INFORMATION

The authors received no specific funding for this work.

## CONFLICT OF INTEREST STATEMENT

The authors declare no conflicts of interest.

## Data Availability

Data sharing is not applicable to this article as no new data were created or analysed.
